# New Insights on NLRP3 Inflammasome: Mechanisms of Activation, Inhibition, and Epigenetic Regulation

**DOI:** 10.1007/s11481-024-10101-5

**Published:** 2024-02-29

**Authors:** Triveni kodi, Runali Sankhe, Adarsh Gopinathan, Krishnadas Nandakumar, Anoop Kishore

**Affiliations:** Department of Pharmacology, Manipal College of Pharmaceutical Sciences, Manipal Academy of Higher Education, Manipal, Karnataka 576104 India

**Keywords:** NLRP3 inflammasome, Epigenetic, DNA methylation, Histone modifications, MicroRNAs, CNS disorders

## Abstract

**Abstract:**

Inflammasomes are important modulators of inflammation. Dysregulation of inflammasomes can enhance vulnerability to conditions such as neurodegenerative diseases, autoinflammatory diseases, and metabolic disorders. Among various inflammasomes, Nucleotide-binding oligomerization domain leucine-rich repeat and pyrin domain-containing protein 3 (NLRP3) is the best-characterized inflammasome related to inflammatory and neurodegenerative diseases. NLRP3 is an intracellular sensor that recognizes pathogen-associated molecular patterns and damage-associated patterns resulting in the assembly and activation of NLRP3 inflammasome. The NLRP3 inflammasome includes sensor NLRP3, adaptor apoptosis-associated speck-like protein (ASC), and effector cysteine protease procaspase-1 that plays an imperative role in caspase-1 stimulation which further initiates a secondary inflammatory response. Regulation of NLRP3 inflammasome ameliorates NLRP3-mediated diseases. Much effort has been invested in studying the activation, and exploration of specific inhibitors and epigenetic mechanisms controlling NLRP3 inflammasome. This review gives an overview of the established NLRP3 inflammasome assembly, its brief molecular mechanistic activations as well as a current update on specific and non-specific NLRP3 inhibitors that could be used in NLRP3-mediated diseases. We also focused on the recently discovered epigenetic mechanisms mediated by DNA methylation, histone alterations, and microRNAs in regulating the activation and expression of NLRP3 inflammasome, which has resulted in a novel method of gaining insight into the mechanisms that modulate NLRP3 inflammasome activity and introducing potential therapeutic strategies for CNS disorders.

**Graphical Abstract:**

## Introduction

### Converging Pathways in Neuroinflammation

Neurodegenerative disorders, which are defined by the progressive loss of neuron structure and function, can affect both the central and peripheral nervous systems. Examples of neurodegenerative disorders such as Alzheimer’s disorder (AD), Multiple Sclerosis (MS), Parkinson’s disorder (PD), motor neuron disease, etc (Hung et al. 2020). In these disorders, apart from the loss of function of the systems that are directly associated with the damaged neurons, marked abnormalities in emotions, thoughts, and behaviors, and are also observed (Hong et al. 2016). As per a recent estimate, neurological disorders including neurodegenerative disorders are now the second-highest cause of mortality (Feigin et al. 2019). It has been known that neuroinflammation is the root cause of neurological conditions such as AD, Schizophrenia, and PD (Kip and Parr-Brownlie 2023; Song et al. 2017). Neuroinflammation, triggered by factors such as trauma, infections, etc., is an innate immune response that involves the activation of microglia, astrocytes, and blood-borne immune cells. Pro-inflammatory cytokines and reactive oxygen species are secreted by activated immune cells (Tohidpour et al. 2017; Welcome 2020). Generally, inflammation has a protective role in tissue injury and repair; however, uncontrolled and chronic neuroinflammation damages the neuronal tissues resulting in neuronal dysfunction and degeneration (Abo-ouf et al. 2013).

A set of multi-protein complexes known as the inflammasomes, that are distributed in the cytoplasm also act as important mediators of neuroinflammation (Bulté et al. 2023; Eren and Özören 2019; Albornoz et al. 2018; Duan et al. 2020). Nucleotide-binding oligomerization domain leucine-rich repeat and pyrin domain-containing protein 3 (NLRP3) inflammasome is currently receiving attention in neurological disorders (Singhal et al. 2014; Guan and Han 2020; Eren and Özören 2019). NLRP3 inflammasome is abundantly expressed in the central nervous system (CNS) where it is triggered by pathogen-associated molecular patterns (PAMPs) and damage-associated molecular patterns (DAMPs) leading to dysregulation of the cellular microenvironment. The NLRP-3 inflammasome stimulates pro-inflammatory caspase-1 that initiates secondary inflammatory reactions leading to neuronal injury (Song et al. 2017). Strategies to regulate NLRP3 inflammasome to control neuronal inflammation are gaining popularity. Numerous studies in animal models indicated the efficacy of NLRP3 inhibitors in the mitigation of diseases associated with NLRP3 overexpression. However, their treatment approach in patients is not yet determined. The NLRP3 inflammasome is linked to inflammatory and neurological disorders due to its inappropriate activation. Hence targeting the NLRP3 inflammasome in different ways such as directly or indirectly inhibiting the NLRP3 inflammasome components, suppressing the NLRP3 inflammasome activations canonical and non-canonical pathways and regulating the epigenetic mechanisms will helps in minimizing the severity of diseases.

The epigenetic processes such as DNA methylation, histone alterations, and microRNAs (miRNAs) are either directly or indirectly related to the formation and control of NLRP3 inflammasome components (Raneros et al. 2021). Targeting these epigenetic mechanisms could be a valuable strategy for restoring inflammasome homeostasis and maintaining a balance between inflammasome function in consequence to environmental signals and the prevention of tissue damage caused by their uncontrolled activation (Poli et al. 2020). Understanding these mechanisms accentuates the potential utility of epigenetic treatments in NLRP3 inflammasome-associated disorders.

Here, we provide a review of the methods of NLRP3 inflammasome induction by canonical, non-canonical, and alternative pathways. We have also presented an update on the effects of NLRP3 inhibitors in NLRP3-associated diseases *in-vitro* and *in-vivo*. Further, to open vistas in therapeutics of NLRP3-driven disease, we have also focused on epigenetic mechanisms to regulate and modulate the NLRP3 inflammasome components.

## NLR Family- NLRP3 Inflammasome Components and Activation

Innate immune responses are collectively regulated by the peripheral nervous system, neuroendocrine system, and CNS. They provide first line defence against invading infections and subsequently halt inflammation to restore balance. This intricate interplay ensures effective immune control and host well-being (Sternberg 2006). The cells involved in the innate immune are monocytes, macrophages, and neutrophils. These express pattern recognition receptors (PRR) which are used to recognize PAMPs and DAMPs (Singhal et al. 2014; Walsh et al. 2014). PAMPs are foreign molecules in damaged tissues, whereas DAMPs are misfolded proteins, aggregated peptides, or mislocated nucleic acids found in the tissues. Although the primary role of PRR is to protect the host from dangerous stimuli, aberrant activation of PRR contributes to the chronic inflammatory process (Banjara and Ghosh 2017; Duan et al. 2020). PRR can be membrane-bound Toll‐like receptors (TLRs), Cytosolic nucleotide oligomerization domain (NOD)‐like receptors (NLRs), C‐type lectin receptors (CLRs), and retinoic acid‐inducible gene I (RIG‐I) ‐like receptors (RLRs) (Saresella et al. 2016; Moretti and Blander 2021). Among these, the NLRs protein family contains 22 human proteins and at minimum of 34 proteins in mice (He et al. 2016a; Freeman and Ting 2016; Yu et al. 2021). These are NLRP1b, NLRP2, NLRP3, NLRC4, NLRC5, NLRP6, NLRP7, NLRP9b, NLRP14, and NLRP12 as well as Absent in melanoma 2 (AIM2) which is a non-NLR inflammasome receptor (Zhou et al. 2016b; Jo et al. 2016; Poudel and Gurung 2018; Freeman and Ting 2016).

Compared to NLRP3, other members of the NLR family (NLRP1b, NLRP2, NLRC4, NLRC5, NLRP6, NLRP7, NLRP9b, NLRP14, and NLRP12) have not been extensively studied in the field of neuroinflammation. And, unlike NLRP3, their assembly processes are less characterized and established due to the lack of structural data (Lechtenberg et al. 2014; Yang et al. 2019b). However, it may be possible that they could modulate inflammatory responses, especially the neuroinflammation. There is preliminary evidence of several NLRs family members role in the adaptive immune system. However, extensive research is needed to support these findings.

The stimulation of these NLRs consequence in the assembly and activation of cytosolic protein complexes known as inflammasomes (Singhal et al. 2014). In addition, it also increases the downstream triggering of nuclear factor kappa light chain enhancer of activated B cells (NF-kB) signaling this head the secretion of inflammatory mediators, cytokines, and chemokines (Banjara and Ghosh 2017). The significance of NLRP3 inflammasome in activating adaptive immune system in response to bacterial, fungal and virus has been demonstrated. NLRP3 is the most predominant and well-studied protein among NLRs family and serves as a prototype for canonical and non-canonical pathways (Sandall et al. 2020).

NLRs family is characterized by their tripartite structure. All NLRs contain caspase activation and recruitment or pyrin domain (CARD or PYD) at the N-terminal, central nucleotide-binding oligomerization domain (NACHT) and C- terminal domain leucine-rich repeats (LRR) which interact with other proteins and promotes the formation of the inflammasome (Haque et al. 2020; Mamik and Power 2017; Hong et al. 2019).

The cryo-electron microscopy reveals that decameric structure of the inactive NLRP3 is in fact the homodimeric assembly of intertwined LRR domains that form pentamers. An LRR transition segment is responsible for the maintaining the molecular contacts between the two pentamers. In addition, this structure also contains the NACHT domain and a pyrin domain dimer. Certain specific inhibitors of NLRP3, like CRID3, stabilizes the structure by binding to the Walker A motif of the nucleotide-binding domain of NLRP3. An understanding of these sites have opened the possibilities for specific targeting of NLRP3 inflammasome. (Hochheiser et al. 2022).

**Fig. 1:**
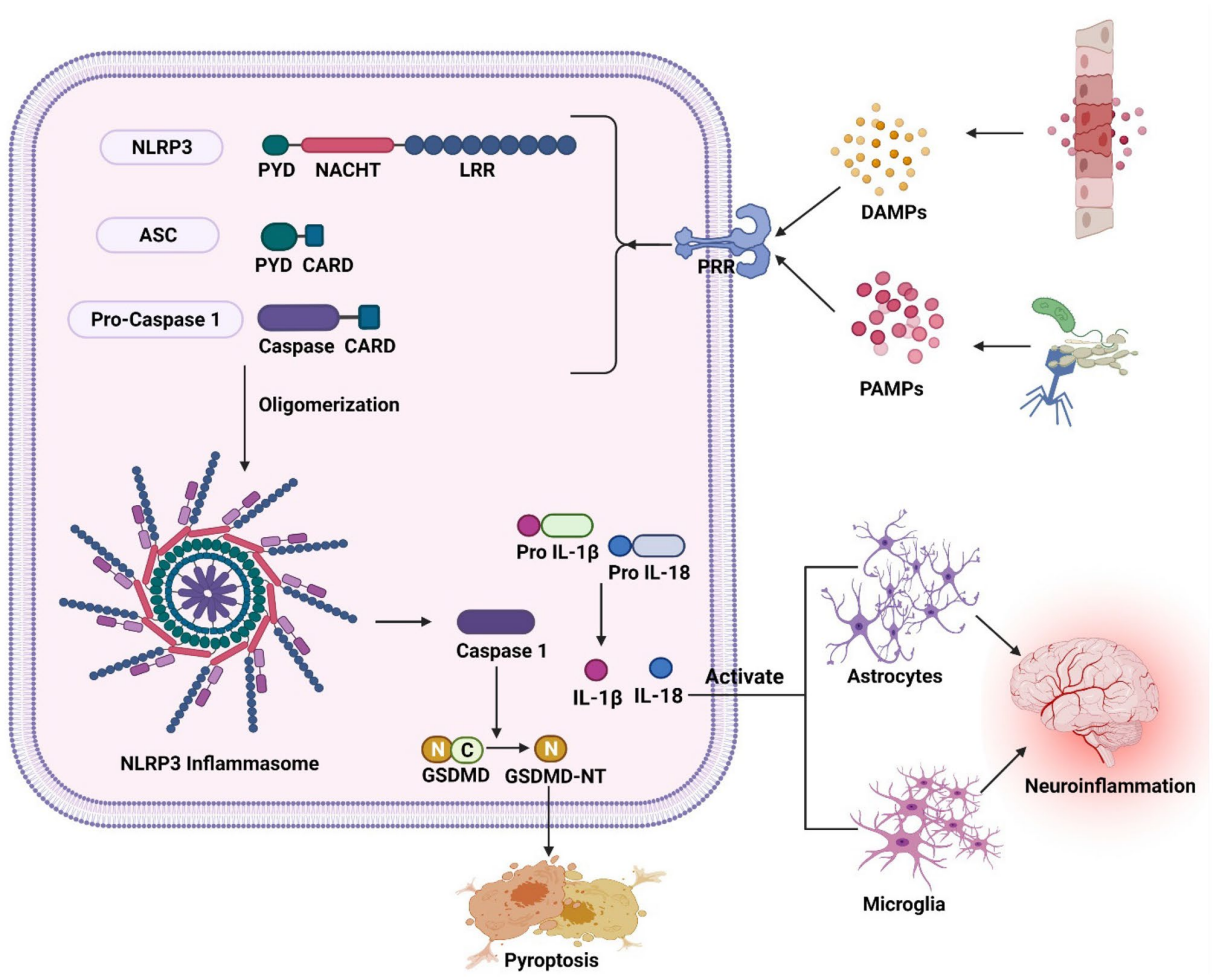
Schematic illustration of the structure of NLRP3 inflammasome and its activation: The detection of PAMPs and DAMPs by PRR causes the assembly of NLRP3, ASC, and procaspase 1, which results in the activation of the NLRP3 inflammasome. The activated NLRP3inflammasome releases caspase-1 that leads to the maturation of IL-1 $\upbeta$ and IL-18 from their inactive forms. These IL-1 $\upbeta$ and IL-18 bind to their receptors on astrocytes and microglia and activate them thus finally leading to neuroinflammation. In addition, the caspase-1 converts the gasdermin D to gasdermin NT which results in pyroptosis. PAMPs: pathogen-associated molecular patterns; DAMPs: damage-associated molecular patterns; PRR: pattern recognition receptors; NLRP3: nucleotide-binding oligomerization domain leucine-rich repeat and pyrin domain-containing protein 3; PYD: pyrin domain; NACHT: nucleotide-binding oligomerization domain; LRR: leucine-rich repeats; ASC: adaptor apoptosis-associated speck-like protein; CARD: caspase activation and recruitment domain; GSDMD: gasdermin D; GSDMD-NT: N-terminal domain of gasdermin D

An inflammasome is formed by three components: a sensor molecule (member of PRRs), an adapter molecule (adaptor apoptosis-associated speck-like protein (ASC) carrying a caspase-activation and recruitment domain), and an effector component (Caspase-1) (Duan et al. 2020). The NLRP3 inflammasome comprises of 1) the cytosolic sensor complex NLRP3 which is a tripartite protein comprising PYD at N- the terminal, the central NACHT domain, and the LRR at C-terminal. 2) Adaptor protein ASC contains PYD at N-terminal and CARD at C- the terminal. 3) Cysteine protease procaspase 1 contains caspase 1 and CARD. NOD with the ATPase activity is important for protein self-oligomerization. Through the homotypic association of the pyrin-pyrin domain interaction, ASC binds to NLRP3 which leads to ASC dimer assembly into a speck-like structure. ASC interconnects with procaspase 1 through the CARD domain which results in the oligomerization of caspase 1 and conversion of inactive pro-enzyme into the active form of caspase 1. Caspase 1 assists in pyroptosis and activation of interleukin-$1\upbeta$& interleukin-18 (Bulté et al. 2023; Sun et al. 2022; Song et al. 2017; Eren and Özören 2019; Xiao et al. 2020) (Fig 1). A recent study has shown that the LRR domain works as a sensor and causes autoinhibition of the NLRP3 by folding back into the NACHT domain (Xiao et al. 2020).

In CNS, NLRP3 inflammasome is predominantly involved in microglia and astrocytes. Microglia are immune cells essential for immune response in the CNS and astrocytes are glial cells that support the neurons. The microglia and astrocytes activate the NLRP3 inflammasome in response to several signals including as misfolded proteins, injured neurons, and cellular debris. Once activated, microglia or astrocytes generate pro-inflammatory cytokines and chemokines, contributing to neuroinflammation. Dysregulation of the NLRP3 inflammasome in microglia and astrocytes have been implicated in several neuronal disorders, where sustained inflammation can lead to neuronal damage. NLRP3 inflammasome mediated microgliosis and astrogliosis are observed in both *in-vitro* and *in-vivo* studies. (Freeman et al. 2017; Scholz and Eder 2017). Astrocytes play a role in enhancing neuroinflammation via the NLRP3 pathway in APP/PS1, and inhibition of NLRP3 inflammasome offers neuroprotection in AD (Nassar et al. 2022; Duan et al. 2021). In chronic mild stress (CMS) mouse model, neuronal defect, behavioural abnormalities, and the development of neurotoxic A1-like astrocytes are all associated with microglial NLRP3 (Li et al. 2022c). In mature oligodendroglia, hyperactivation of Drp1 inhibited hexokinase 1 leading to glycolytic defects that trigger NLRP3 inflammasome in AD models, whereas knockout of Drp1 corrected glycolytic defect, decreased activation of NLRP3 inflammasome, decreased myelin and axonal loss, and also enhanced cognitive function in AD models (Zhang et al. 2020a).

Inflammasome-mediated neuroinflammation may influence neural development, by generating inflammatory mediators that affect neurogenesis, synaptogenesis, and neural system formation. The specific processes and pathways of regulation of NLRP3 inflammasome during neural development are currently under investigation. The mutations in the NLRP3 gene are associated with the familial cold autoinflammatory syndrome, muckle wells syndrome, neonatal-onset multisystem inflammatory disease, chronic infantile neurological cutaneous, and articular syndrome. Although the NLRP3 inflammasome is controlled, mutations in this gene that result in inflammasome overactivation have been found in autoinflammatory diseases (Eren and Özören 2019).

R262W, L307P, and V200M are specific point mutations in NALP3 gene, also known as cold-induced autoinflammatory syndrome 1 gene (CIAS1), and pyrin-containing Apaf-1–like protein (PYPAF1) are associated with familial cold urticaria (FCU)/ familial cold autoinflammatory syndrome (FCAS) and Muckle-wells syndrome (MWS). The mutations in the NALP3/CIAS1/PYPAF1 genes offers healthcare professionals a novel method for disease diagnosis and could help in drug development for certain autoinflammatory diseases (Aganna et al. 2002; Gattorno et al. 2007). More than 90 genetic variants of the NLRP3 gene, most of which are autosomal dominant missense point mutations in exon 3 that codes for the NATCH domain, have been related to the characteristic symptoms of cryopyrin-associated periodic syndrome (CAPS) (Touitou et al. 2004; Masters et al. 2009). Exon 3 of the NLRP3 gene knock-in mice models with the point mutations R258W and A350V, which are equivalent to the human NLRP3 R260W and A352V mutations respectively, are linked to MWS. And, mutations in CAPS patients are reported to be located on exon 3 (Naz Villalba et al. 2016). Even without an NLRP3 agonist, the abnormal inflammasome structural alignment and hyperactivation caused by R258W and A350V mutations cause spontaneous IL-1 release. (Brydges et al. 2009; Meng et al. 2009; Conforti-Andreoni et al. 2011). The LRR domain encoded by exon 6 and the Y859C mutations have been linked to a distinct CAPS phenotype and late-onset MWS traits (Jéru et al. 2010). Similarly, another variant, Q703K, is involved in autoinflammatory diseases (Theodoropoulou et al. 2020).

### Mechanism of NLRP3 Inflammasome Activation

The major role of NLRP3 inflammasome is after detecting endogenous and exogenous stress signals it translates them into inflammatory reactions (Herman and Pasinetti 2018; Hong et al. 2019). Hence, the NLRP3 inflammasome is a crucial mediator of neuroinflammation (Long et al. 2019). It has been known that NLRP3 detects changes in cellular stress (Pellegrini et al. 2019). At present, two modes have been characterized for NLRP3 activation: the canonical and non-canonical pathways (Guan and Han 2020).

#### Activation of Canonical NLRP3 Inflammasome Pathway

The canonical NLRP3 inflammasome activation involves priming and activation (Mangan et al. 2018; Kelley et al. 2019; Song et al. 2017).

**Fig. 2:**
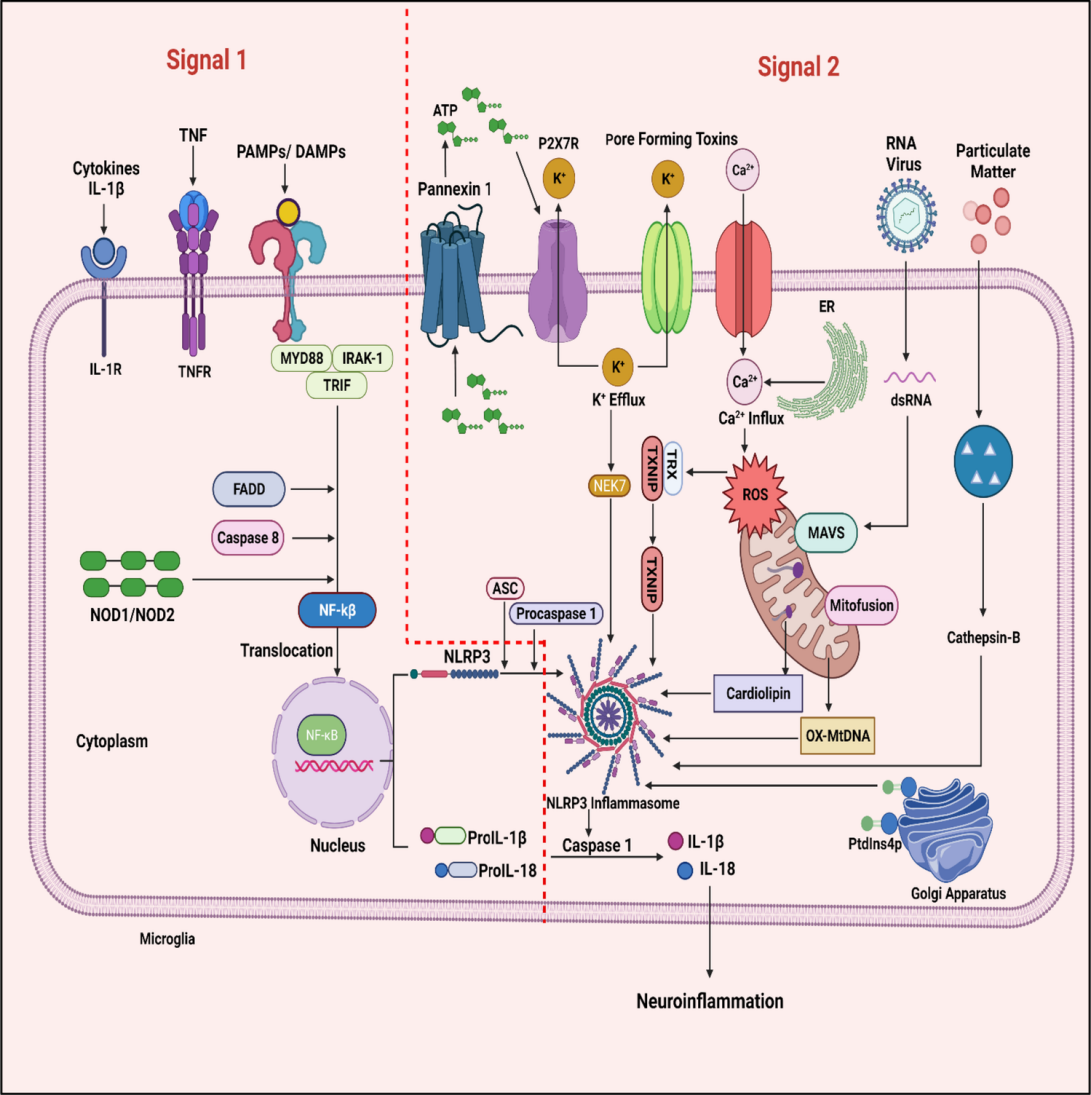
The overview of different cellular mechanisms involved in canonical NLRP3 inflammasome activation: This is regulated by the two signals. Signal 1(left side) is a priming signal initiated by the PRR family by detecting the microbial components or ATP and resulting in the involvement of myddasome complex. This myddasome complex activates the NF-kB by translocating into the nucleus. Activated NF-kB further upregulates NLRP3 and pro-IL-1β. Signal 2 (right side) is an activation signal produced by stimuli like pore-forming bacterial, fungal, and viral toxins, extracellular ATP, Particulate matter, and cellular events that are shown to assemble and activate the NLRP3 inflammasome. TNF: tumor necrosis factor; TNFR: tumor necrosis factor receptor; PAMPs: pathogen-associated molecular patterns; DAMPs: damage-associated molecular patterns; MyD88: myeloid differentiation primary response 88; IRAK-1: interleukin-1 receptor-associated kinase 1; TRIF: TIR-domain-containing adaptor-including interferon-β; FADD: fas-associated protein with death domain; NF-kB: nuclear factor kappa light chain enhancer of activated B cells; ATP: adenosine triphosphate; P2X7R: P2X purinergic receptor 7; NEK7: NIMA-related kinase 7; NLRP3: nucleotide-binding oligomerization domain leucine-rich repeat and pyrin domain-containing protein 3; ASC: adaptor apoptosis-associated speck-like protein; TXNIP: thioredoxin interacting protein; ROS: reactive oxygen species; MAVS: mitochondrial antiviral-signaling protein; OX-MtDNA: oxidized mitochondrial deoxyribonucleic acid

Priming /Transcriptional signal: It is induced through PRR families such as TLRs, or NLRs or through cytosolic receptors which recognize PAMPs (microbial components like lipopolysaccharide (LPS)) and DAMPs (adenosine triphosphate (ATP) and particulate matter) resulting in the transcriptional factor activation NF-kB by translocation into the nucleus via myddasome complex (Söderbom and Zeng 2020; Mangan et al. 2018). This complex includes Myeloid differentiation primary response 88 (MyD88), Interleukin-1 receptor-associated kinase 1 (IRAK-1), TIR-domain-containing adaptor-including interferon-β (TRIF), Fas-associated protein with death domain (FADD) and Caspase-8 are essential for activation of NF-kB and subsequent upregulation of NLRP3 and pro-IL-1β (Kelley et al. 2019; Chen et al. 2023; Cao et al. 2023). In addition**,** the transcriptional signal also regulates the activation of NLRP3 at post-transcriptional modifications (He et al. 2016a) (Fig 2).

Activating signal: It is induced through PAMPs stimuli such as pore-forming bacterial, fungal, and viral toxins, DAMPs such as extracellular ATP, Particulate matter such as asbestos, silica, uric acid crystals, alum, amyloid- β fibrils, cholesterol, and calcium crystals results in NLRP3 inflammasome assembly and activation (Voet et al. 2019; Lamkanfi and Dixit 2012; Huang et al. 2021; Zheng et al. 2023). It is reported that the activating signal preceding the priming signal, will not trigger the activation of NLRP3 inflammasome (Herman and Pasinetti 2018). However, there are certain studies that suggested priming signal may not be required for NLRP3 inflammasome activation. This indicates that in some situations specific triggers may immediately activate the inflammasome without the need for prior priming step. Human monocytes exhibit the ability to form canonical NLRP3 inflammasomes without the need for priming. The activation of NLRP3 by the inducer- nigericin triggers the processing and release of constitutively expressed IL-18, even in the absence of priming. This process is reliant on K+ and Cl- efflux, leading to ASC oligomerization, cleavage of caspase-1, and gasdermin D (GSDMD) cleavage. The release of IL-18 is modulated by factors such as the NLRP3 inhibitor MCC950, NLRP3 deficiency, and GSDMD deficiency, implicating pyroptosis as the underlying mechanism for the release of IL-18. This unique behaviour of unprimed human monocytes highlights their distinct role in the inflammatory response through NLRP3 inflammasome activation without any priming step (Gaidt et al. 2016; Gritsenko et al. 2020). Ionic fluxes, mitochondrial dysfunction, reactive oxygen species, and lysosomal disruption are considered cellular events found to upregulate the NLRP3 inflammasome stimulation (Pellegrini et al. 2019).

Ionic flux: Potassium (K^+^) efflux, calcium (Ca^2+^) mobilization, chlorine (Cl^−^) efflux, and sodium (Na^+^) influx are some of the ionic fluxes triggered by various NLRP3 stimuli that result in the engagement of NLRP3 inflammasome (Pellegrini et al. 2019). Among these K^+^ efflux is a dominant cellular event induced by the binding of extracellular ATP or nigericin with P2X purinergic receptor 7 (P2X7). Activation of the ligand-gated ion channel, P2X7 receptor, results in the opening of K^+^ efflux channels (Wang et al. 2019b; Eren and Özören 2019; Próchnicki et al. 2016). In addition, bacterial toxins create pores in the cell membrane which promote K^+^ efflux (Próchnicki et al. 2016). The reduction in intracellular K^+^ levels has been proposed as an important event that stimulates NLRP3 inflammasome (Moretti and Blander 2021; Yu et al. 2021). K^+^ efflux activates NIMA-related kinase 7 (NEK7) in the cytoplasm, which interacts with the LRR domain of NLRP3 and begins the NLRP3 inflammasome assembly (Wang et al. 2022c; Herman and Pasinetti 2018; He et al. 2016b). Excess Ca^2+^ released from the endoplasmic reticulum (ER) induces mitochondrial Ca^2+^ accumulation and damage, resulting in the generation of mtROS and release of oxidized mtDNA, which activate NLRP3 inflammasome (Li et al. 2021a). A study revealed that Ca^2+^ channel inhibition in the ER can decrease caspase-1 activation (Murakami et al. 2012). In addition, lysosomal dysfunction can induce Ca^2+^ mobilization and K+ efflux (Li et al. 2021a). Increased cytosolic Ca^2+^ encourages the interaction of NLRP3 with ASC through an unknown mechanism (Murakami et al. 2012). NLRP3 activation stimuli such as ATP and nigericin promote the Cl^−^ efflux by enrichment of chloride intracellular channels (CLIC) and volume-regulated anion channels (VRAC) (Tang et al. 2017).

Mitochondrial dysfunction and ROS: Mitochondrial dysfunction occur due to a variety of NLRP3 stimuli. The signals derived from mitochondrial dysfunction are oxidized mitochondrial deoxyribonucleic acid (mtDNA) as well as mitochondrial reactive oxygen species (mtROS) known to drive the NLRP3 inflammasome (Zhou et al. 2011; Shimada et al. 2012). Further, the three mitochondrial proteins- mitofusin-2, cardiolipin, and mitochondrial antiviral-signaling protein (MAVS) expressed during mitochondrial stress or RNA viral infections are believed to stimulate NLRP3 inflammasome (Iyer et al. 2013; Park et al. 2013). A study reported that disturbance of complex I and complex III of the mitochondrial respiratory chain produces mtROS and activates NLRP3 (Zhou et al. 2011). Mitophagy is a major regulator for NLRP3 activation as it lowers mtROS (Zhou et al. 2011). ROS generation promotes thioredoxin interacting protein (TXNIP) detachment from TRX and enhances the TXNIP-NLRP3 binding which upregulates the stimulation of NLRP3 inflammasome and there after maturation of proinflammatory mediators (Ye et al. 2017).

Several studies have revealed the link between the mitochondrial electron transport chain (ETC) and NLRP3 activation (Groß et al. 2016; Nakahira et al. 2011; Zhou et al. 2011). Mitochondrial ETC plays a critical role in maintaining PCr-dependent release of ATP levels and the increased levels of ATP activate NLRP3 inflammasome. Blocking of mitochondrial ETC complex I, II, III and V supressed the activation of NLRP3 inflammasome by reducing PCr and ATP levels, independent of ROS pathway (Billingham et al. 2022). However, there are also reports of mitochondrial ETC mediated modulation of NLRP3 inflammasome via ROS pathway (Holley and Schroder 2020; Neuwirt et al. 2021). Loss of function of NLRP3 inflammasome influenced by the inhibition of mitochondrial ETC could be reversed (Seo et al. 1998; Sommer et al. 2020; El-Khoury et al. 2013) (Billingham et al. 2022). Depletion of mtDNA is also factor that reduces mitochondrial ETC function and lowers ATP levels. mtDNA activates NLRP3 inflammasome via the involvement of cyclic GMP-AMP synthase (cGAS) and the stimulator of interferon genes (STING) pathways. Also, eliminating mtDNA via transcription factor A mitochondrial (TFAM) ablation impairs the NLRP3 inflammasome activation, possibly due to decreased mitochondrial ATP (Zhong et al. 2018; Billingham et al. 2022).

Lysosomal disruption: Disruption of lysosomal membranes due to certain phagocytosed particulate matter ends in the secretion of lysosomal components into the cytoplasm (Zhang et al. 2020c; Zhou et al. 2016a). Cathepsin B released from the lysosome encourages NLRP3 inflammasome (Zhang et al. 2020c). Depletion of zinc levels leads to lysosomal rupture and promotes NLRP3 inflammasome (Jo et al. 2016) (Fig 2).

#### Noncanonical NLRP3 Inflammasome Pathway

This pathway is independent of TLR4 signaling and dependent on caspase 4 and 5 in humans and caspase 11 in mice (Mangan et al. 2018). LPS activates caspase 11 which in turn triggers pannexin 1 channel opening. ATP enters through the channel and promotes k+ efflux that upregulates NLRP3 inflammasome as well as simultaneous secretion of IL-1β and IL-18 (Kelley et al. 2019; Shi et al. 2015). In addition, activation of caspase 11 induces pyroptosis by the breakdown of GSDMD. The N-terminal domain of gasdermin D (GSDMD-NT) can induce pores on the membranes and boosted the assembly of NLRP3 inflammasome (Wang and Hauenstein 2020; Ding et al. 2016a; Liu et al. 2016; Accogli et al. 2023).

#### Alternative NLRP3 inflammasome Pathway

Besides canonical and non-canonical pathways, there is a recent NLRP3 inflammasome pathway found in monocytes of humans and is independent of K+ efflux, with an absence of pyroptosis (Starobova et al. 2020). This pathway is activated by LPS and depends on TLR4, caspase 8, FADD, and receptor-interacting serine/threonine-protein kinase 1 (RIPK1) that triggers NLRP3 inflammasome assembly (Yang et al. 2019d; Gaidt et al. 2016). Alternative inflammasome is occurred in human and porcine monocytes, however not seen in murine cells (Gaidt et al. 2016; Yang et al. 2019d). This pathway depends on TLR4–TRIF–RIPK1–FADD–CASP8 signaling to enhance NLRP3 activation. Moreover, this signaling is restricted to an alternative inflammasome, it has no role in classical NLRP3 inflammasome (canonical and non-canonical) (Gaidt et al. 2016).

Besides LPS, apolipoprotein C3 (ApoC3) has exhibited NLRP3 inflammasome activation via an alternative pathway in human monocytes, by building a heterotrimer among Toll-like receptors- TLR2, TLR4, and SLP adaptor and CSK interacting membrane protein (SCIMP). This heterotrimer triggers Lyn/Syk-dependent calcium entry and the formation of reactive oxygen species, which activates caspase-8 (Zewinger et al. 2020). Also, ApoC3 activates NLRP3 inflammasome by increasing the binding of TXNIP to NLRP3 in the presence of ROS (Zhou et al. 2010).

A study reported that heat killed gram negative bacteria acts as an NLRP3 inflammasome stimulant in human monocytes. The short isoform of cFLIP (cFLIPs), which was activated by NF-κB, negatively regulated caspase-8 and arrested the alternative NLRP3 inflammasome activation in response to heat killed bacteria. When compared to classic inflammasome pathways, this response occurs quickly and results in the release of IL-1β by human monocytes (Gao et al. 2023).

In the context of neurodegenerative disorders, NLRP3 inflammasome activation via canonical pathway has been the most investigated, compared to the non-canonical and alternate pathways. The non-canonical path primarily responds to LPS in the cytosol and is frequently linked with caspase-11/ 4/5. Alternative activation pathway, the least studied one, involves several stimuli and caspases. Therefore, the highlights of the canonical pathway that play a crucial role in neuronal disorders have been a focus of this review.

## Update on NLRP3 Inflammasome Inhibitors in NLRP3 Inflammasome Mediated Diseases

NLRP3 and its downstream pathways are being evaluated as targets for inflammation and autoimmune disorders. NLRP3 inhibitors specifically target the NLRP3 protein that is involved in the inflammatory responses of NLRP3 inflammasome, whereas NLRP3 downstream inhibitors suppress the inflammatory cascade after the NLRP3 activation has occurred. The factor that determines their selection and adoption, depends on the disease or condition that is involved. When NLRP3 inflammasome is directly involved in inflammation, inhibiting the activation of NLRP3 may be the better option. On the other hand, disorders that are triggered by specific downstream pathways will respond to effectively to specific downstream inhibitors.

Even though the efficacy of NLRP3 downstream inhibitors have been established against autoinflammatory conditions, there are no reports on their clinical uses or clinical trials, for neurological disorders. For e.g., Caspase 1 inhibitors like VX-765 and Ac-YVAD-cmk have shown beneficial effects in managing atherosclerosis (Li et al. 2020), cerebral ischemia (Liang et al. 2021) and esophagitis (Wang et al. 2019a). Probably, their ineffective penetration of blood-brain barrier and the accompanied pyroptosis which exacerbates the production of IL-18 and other inflammatory mediators may hamper their efficacy. Therefore, studies on novel molecules that target the NLRP3 inflammasome directly could provide better therapeutic strategies to treat NLRP3-driven diseases (Duan et al. 2020).

Inhibiting the NLRP3 inflammasome ameliorates NLRP3-dependent pathological diseases. Therefore, targeting and suppression of NLRP3 inflammasome offer promising therapeutic applications. Here, we list the known therapeutic inhibitors of NLRP3 inflammasome and respective disease targets. The specific NLRP3 inflammasome inhibitors are MCC950, POPs, CY-09, OLT1177, Tranilast, Oridonin, Bot-4-one, 3,4-methylenedioxyβ-nitrostyrene (MNS), Glyburide, and JC-171 and the non-specific inhibitors include BAY-117082, Parthenolide, Beta-hydroxybutyrate** (**BHB), NSAIDs and BTK.

MCC950: Also called CRID3 or CP-456,773 is a highly specific reversible inhibitor of NLRP3, that prevents the oligomerization by binding to active as well as inactive NACHT domains of NLRP3 and inhibiting the hydrolysis of ATP to ADP required for the oligomerization (Coll et al. 2015). It keeps the NLRP3 in an inactive state (Tapia-Abellán et al. 2019). This small molecule suppresses canonical and noncanonical activation pathways that induce NLRP3 inflammasome (Coll et al. 2015; Starobova et al. 2020). MCC950 has been found in reducing the progression of neurological diseases by suppressing the NLRP3 inflammasome. It was observed that MCC950 inhibited NLRP3 inflammasome and improved cognitive function by promoting the clearance of β-amyloid plaques in APP/PS1 mice (Dempsey et al. 2017; Duan et al. 2020), ameliorated dopaminergic neurodegeneration in α-synuclein fibril induced inflammasome activation in mice (Gordon et al. 2018), and reduced dementia via blocking α-synuclein accumulation in the hippocampus of mice (Ren et al. 2022). It also improved neurological outcomes by reducing neuroinflammation in traumatic brain injury (TBI) models in mice (Fan et al. 2018; Ismael et al. 2018; Xu et al. 2018). Treatment with MCC950 protected against subarachnoid hemorrhage-mediated brain injury (Luo et al. 2019), cerebral ischemia/reperfusion-induced neuronal ferroptosis via reducing ROS generation (Wu et al. 2023) as well as nerve injury after intracerebral hemorrhage in rats (Guo et al. 2022). MCC950 declined the MS in the experimental autoimmune encephalomyelitis (EAE) model in mice by suppressing the production of IFN-γ and IL-17 (Coll et al. 2015; Freeman and Ting 2016). In addition, arresting NLRP3 inflammasome by administration of MCC950 blocked Isoproterenol-induced cardiac dysfunction by suppressing cardiomyocyte senescence (Shi et al. 2022) and ameliorated heart failure (HF) in obese mice by improving the cardiometabolic dysfunction (Wang et al. 2022a). MCC950 lowered doxorubicin-induced myocardial injury by repressing NLRP3-mediated pyroptosis in *in-vivo* and *in-vitro* models (Zhang et al. 2021b) as well as alleviated heart failure-induced ventricular arrhythmia (Jiang et al. 2022). In apoE^−/−^ mice fed with high-fat and THP-1-derived macrophages, MCC950 attenuated atherosclerosis via hindering macrophage pyroptosis and IL-1β and IL-18 production (Zeng et al. 2021). MCC950 showed an anti-pyroptosis effect by suppressing gasdermin D and improved Duchenne muscular dystrophy (Dubuisson et al. 2022). MCC950 relieved acute pancreatitis in rats by significantly decreasing the pancreatic edema, necrosis, and inflammatory factors (Wang et al. 2022d) and minimizing the pathological damage to the pancreas and intestine in mice (Shen et al. 2022). In CCl_4_-induced acute liver injury in mice (ALI), MCC950 minimized ALI via enhancing macrophage polarization and myeloid-derived suppressor cell function (Yan et al. 2021). In an *in-vitro* study, LPS remarkably suppressed the viability of human periodontal ligament cells (HPDLCs), increases proinflammatory cytokines, and lowered osteogenic gene expression. While co-administration of MCC950 suppressed proinflammatory cytokines and boosted the osteogenic gene in HPDLCs (Peng et al. 2021). However, in streptozotocin-induced diabetic mice, MCC950 showed adverse effects on the renal system by increasing renal inflammation, mesangial expansion, and glomerulosclerosis (Østergaard et al. 2022). MCC950 is a promising molecule that directly abolishes the assembly of NLRP3 inflammasome (Herman and Pasinetti 2018). MCC950's focus on NLRP3 inflammasome inhibition offers a novel therapeutic approach for a variety of diseases. However, the phase II clinical trials using MCC950 for rheumatoid arthritis were discontinued following reports of liver damage (Mangan et al. 2018).

PYD-Only Proteins (POPs): The PYD-only proteins POP1 and POP2 binds to ASC and prevent the PYD-PYD interactions between NLRP3 and ASC that eventually decline the activation of caspase-1 (Schroder and Tschopp 2010; Stehlik and Dorfleutner 2007; Dorfleutner et al. 2007; Zhang et al. 2020c). Pops are present in humans and higher primates (Devi et al. 2020). Decreased POP1 levels in CAPS patients are inadequate to prevent overactive inflammasome activity (de Almeida et al. 2015). However, POP2 suppresses both priming and activation of NLRP3 inflammasome (Ratsimandresy et al. 2017). Both POP1 and POP2 can suppress NF-kB activation thereby obstructing the initial signal of inflammasome activation (Bedoya et al. 2007; Stehlik and Dorfleutner 2007; Atianand and Harton 2011). Unlike POP1 and POP2, POP3 is stimulated by dsDNA viruses and binds to PYDs of the DNA sensors AIM2 and IFI16. POP3 appears to specifically suppress AIM2-like receptor inflammasomes (Khare et al. 2014). While POP4 is enhanced by stimulation of LPS and inhibits NF-kB activity by suppressing TLR-induced RelA/p65 transactivation (Porter et al. 2014; Lara-Reyna et al. 2022).

CY-09: NLRP3 ATPase activity is crucial for its oligomerization and activation (Duncan et al. 2007). CY-09 directly interacts with the walker A motif (ATP- binding site) of the NLRP3 NACHT domain and suppresses the ATPase activity, this inhibits the assembly and activation of NLRP3 (Duncan et al. 2007; Jiang et al. 2017). CY-09 possesses potent anti-inflammatory activity due to the regulation of NLRP3 inflammasome and its inhibitory effect is independent of the priming signal or post-translation modifications (Yang et al. 2019d). CY-09 exhibited protective effects of the CAPS as well as type-2 Diabetes in mouse models. Besides it also effectively downregulated NLRP3 in synovial cells of gout patients (Jiang et al. 2017). Furthermore, CY-09 inhibited NLRP3 inflammasome by blocking the ADP and collagen-induced human platelet aggregation (Qiao et al. 2018). It also CY-09 exhibited anti-depressant effects in LPS-induced neuroinflammation in mice via mitigating the neuroinflammation in microglia (Wang et al. 2022b). It was observed that in TNF- α stimulated chondrocytes and destabilization of the medial meniscus (DMM) induced osteoarthritis model, CY-09 had offered a protective effect by regulating extracellular matrix homeostasis and chondrocytes inflammation (Zhang et al. 2021c). CY-09 also reduced hepatic steatosis in NAFLD mice (Wang et al. 2021).

OLT1177: OLT1177 is a β-sulfonyl nitrile molecule, that inhibits NLRP3 inflammasome by binding directly to NLRP3 NACHT domain and hamper ATPase activity which preventing the interaction between NLRP3 and ASC. OLT1177 inhibited canonical as well as non-canonical activation of NLRP3 in an *in-vitro* model followed by decreasing the IL-1β and IL-18 production without suppressing the synthesis of IL-1β precursor protein in isolated monocytes of CAPS patients. Furthermore, humans receiving a high dose of OLT1177 for 8 days have shown no biochemical adverse effects (Marchetti et al. 2018a; Yang et al. 2019d). Human phase II trials of OLT1177 are currently on, for the treatment of degenerative arthritis (Toldo and Abbate 2018). It has long half-life and do not exhibit any significant organ or haematological toxicities. It has a good tolerance and safety margin (Marchetti et al. 2018a). OLT1177 has also reported potential therapeutic benefits in dextran sodium sulfate (DSS)-induced colitis in rodents (Oizumi et al. 2022; Saber et al. 2021), spinal cord injury (SCI) (Amo-Aparicio et al. 2022), myocardial infarction (Aliaga et al. 2021), AD (Lonnemann et al. 2020), MS (Sánchez-Fernández et al. 2019), and acute arthritis (Marchetti et al. 2018b).

Tranilast: Tranilast is first identified as an anti-allergic agent, and treated several inflammatory diseases (Darakhshan and Pour 2015). It acts as an inhibitor of the NLRP3 inflammasome by binding to the NLRP3 NACHT domain and prevents the interaction among NLRP3-NLRP3 and ASC oligomerization (Huang et al. 2018). Tranilast is a clinically approved drug for allergy and is well-tolerated at higher dose levels (Konneh 1998; M et al. 2005) and (Huang et al. 2018). It is known to have therapeutic benefits in NLRP3-mediated diseases in mouse models, such as CAPS, gouty arthritis, and type-2 diabetes (Huang et al. 2018; Yang et al. 2019d). Recent studies reported that tranilast could be utilized as a beneficial and safe adjuvant to boost the effectiveness of anti-viral therapy in COVID-19 patients (Saeedi-Boroujeni et al. 2022) as well as a potential anti-inflammatory drug for COVID-19 (Saeedi-Boroujeni et al. 2021). Moreover, tranilast alleviated gestational diabetes in the genetic mouse model by suppressing inflammatory responses (Cao and Peng 2022).

Oridonin: Oridonin is the main bioactive ingredient of *Rabdosia Rubescens* treated inflammatory diseases (Chen et al. 2009; He et al. 2018; Ma et al. 2011). Oridonin specifically suppresses the NLRP3 inflammasome by binding irreversibly to cysteine 279 of NLRP3 and preventing its interaction with NEK7 (He et al. 2018). Oridonin can repress proinflammatory cytokine secretion, such as TNF-α and IL-6, by blocking MAPK or NF-kB activation (Huang et al. 2005; Xu et al. 2009; Zhao et al. 2017). Importantly, oridonin shows promising therapeutic results in the treatment of Crohn’s disease (Wang et al. 2015), AD (Wang et al. 2014) and cerebral amyloidosis (Zhang et al. 2013), and TBI (Yan et al. 2020). Moreover, by inhibiting the NLRP3 inflammasome, oridonin showed protective effects in peritonitis, type-2 DM, and gout (He et al. 2018). Also, oridonin rescues LPS-stimulated acute lung injury by targeting Nrf2 (Yang et al. 2019a). Several oridonin derivatives are created and evaluated for the treatment of cancer (Liu et al. 2021; Ding et al. 2016b).

Bot-4-one: Bot-4-one possesses anticancer (Kim et al. 2011) and immunomodulatory effects (Lee et al. 2016; Kim et al. 2016a). Bot-4-one is a covalent modifier that abrogates the activation of NLRP3 inflammasome by binding to the NLRP3 NACHT domain and suppressing the ATPase activity. As an NLRP3 alkylator, Bot-4-one enhances the ubiquitination of NLRP3 contributing to the suppression of NLRP3 inflammasome in bone marrow derived-macrophage (BMDMs) and monosodium urate-induced peritonitis mouse model (Shim et al. 2017).

Michael acceptors: These carry a common structure known α, β-unsaturated carbonyl group (Haque et al. 2020) that includes compounds such as Parthenolide, BAY 11–7082, and MNS (Voet et al. 2019).

Parthenolide: Parthenolide is a sesquiterpene lactone that shows an anti-inflammatory effect by declining the expression of NF-kB, caspase-1, and NLRP3 ATPase activity (Mosayebian et al. 2021; Juliana et al. 2010). Unlike others, it can suppress multiple inflammasomes in macrophages by suppressing caspase-1 (Juliana et al. 2010). It ameliorated cystic fibrosis by inhibiting inflammation in mice (Saadane et al. 2007; Wang et al. 2020b) and reduced BBB permeability in a stroke rat model (Dong et al. 2013; Mamik and Power 2017). Additionally, parthenolide relieved LPS-induced inflammation in bv2 microglia cells and OGD-mediated neuronal apoptosis and oxidative stress in HT22 neuronal cells. It also improved neuroinflammation and memory in the TBI mouse model induced by a controlled cortical impact device (Ding et al. 2022). Recent *in-vitro* as well as *in-vivo* models found the pharmacological effect of parthenolide in insulin resistance induced by obesity (Chinta et al. 2022), hepatic fibrosis (Cui et al. 2021), acute hepatitis (Wang et al. 2016), and familial Mediterranean fever (Mosayebian et al. 2021).

BAY11-7082: BAY11-7082 is an inhibitor of IkB kinase β which also hampers ATPase activity by alkylation of cysteine residues at the ATPase region of NLRP3 in macrophages (Juliana et al. 2010; Singh and Jha 2018). BAY11-7082 has beneficial effects in ameliorating psoriasis (Irrera et al. 2017b), diabetic nephropathy (Kolati et al. 2015), EAE (Lang et al. 2022), neuropathic pain in dorsal root ganglions (Zhang et al. 2017), and TBI (Irrera et al. 2017a).

MNS: MNS is a specific NLRP3 inhibitor that interacts with NLRP3 domains NACHT and LRR and suppresses the ATPase activity (Wang et al. 2020b). This blocks oligomerization and speck formation of ASC (He et al. 2014). Recent studies reported that MNS suppresses NLRP3 inflammasome and decreased dextran sulfate sodium (DSS)-induced colitis in mice (Zheng et al. 2022) and fungal pathogen-induced airway inflammation (Patel et al. 2018). It was observed that combinational treatment of MNS and cytokine-induced killer (CIK) cells augmented its anti-tumor effect in pancreatic ductal adenocarcinoma (Liu et al. 2020).

Glyburide: Glyburide is commonly used for treating Diabetes. It additionally inhibits NLRP3 independent of ATP-sensitive K+ plus channels and suppresses IL-1β secretion as well as blocks ASC aggregation (Lamkanfi et al. 2009; Mamik and Power 2017). In mouse models, glyburide effectively blocked NLRP3-dependent diseases and ameliorated LPS-mediated septic shock and bronchopulmonary dysplasia (Liao et al. 2015; Lamkanfi et al. 2009). Glyburide suppressed the bone resorption induced by traumatic occlusion in a rat model (Arita et al. 2020), lowered pathology in *leishmania braziliensis* infection by reducing inflammatory reactions (Carvalho et al. 2020), and inhibited *candida albicans* (Lowes et al. 2020). Furthermore, glyburide improved the diabetic-induced fracture model in mice by decreasing the osteoclasts and expression of IFN-γ, TNF-α, and IL-6 in the fracture calluses (Yang et al. 2019c). In a rat model of chronic bladder outlet obstruction, glyburide enhanced bladder nerve density (Hughes et al. 2019) and attenuated bladder decompensation and fibrosis by inhibiting IL-1β (Hughes et al. 2017; Hughes et al. 2019). Also, glyburide reported therapeutic effects in chronic stress-induced comorbidity of depression-like behaviour and insulin resistance in the mice model (Su et al. 2017), and cerebral ischemic reperfusion injury in the rat model (Teng et al. 2018). Glyburide has effectively hindered the secretion of proinflammatory cytokines in Crohn’s patients’ mucosal explants and IL-10-/- mice (Liu et al. 2017b). In both *in-vitro* (hepatic cells) and *in-vivo* (cecal ligation and puncture model) studies of septic acute liver injury, glyburide attenuated liver injury via suppressing the hepatic cell pyroptosis (Chen et al. 2016c), hindered hepatic stellate cell-mediated fibrosis (Arriola Benitez et al. 2020) and robustly minimized hepatic steatosis-induced by high fructose diet (Singh et al. 2022). However, the dose of glyburide needed to any significant exhibit anti-inflammatory effect results in severe hypoglycaemia, and therefore, it’s use is limited to the management of type 2 diabetes mellitus (DM) (Mangan et al. 2018).

JC-171: A hydroxysulfonamide analog, JC-171 inhibits the NLRP3 inflammasome by suppressing the interaction among NLRP3- ASC and decreased the disease progression of EAE a mouse model of MS. JC-171 suppressed LPS/ATP -induced IL-1β in J774A.1 macrophage, bone-marrow-derived macrophages, and LPS-challenged mice (Guo et al. 2017).

BHB: BHB, is a ketone metabolite that inhibits the NLRP3 activation by blocking ASC oligomerization and K+ efflux. BHB ameliorated caspase-1 activation and IL-1β release in NLRP3-dependent diseases such as familial cold autoinflammatory syndrome, Muckle-wells syndrome, and, urate crystal-mediated peritonitis (Youm et al. 2015; Kaufmann et al. 2017). Intriguingly, deactivating NLRP3 inflammasome by BHB lowered osteolysis via repressing osteoclast differentiation and function (Wu et al. 2022), alleviated cisplatin-induced acute kidney injury in mice and human proximal tubular epithelial cell line (HK-2) (Luo et al. 2022b), mitigated anxiety in post-traumatic stress disorder rodent model (PTSD) (Yamanashi et al. 2020), protected against CUS-induced depressive- and anxiety-related behaviors (Yamanashi et al. 2017) and depression (Kajitani et al. 2020). Besides, in an *in-vitro* model, BHB reversed the LPS/ATP-induced C6 glioma cell migration by lowering caspase-1 and IL-1β (Shang et al. 2018). BHB reduced the acetoacetate-stimulated NLRP3 inflammasome and IL-1β secretion in bovine peripheral blood mononuclear cells (Onizawa et al. 2022). BHB penetrates BBB and shows neuroprotective properties (Yang et al. 2021).

NSAIDs: They exhibit a suppressive effect on NLRP3 by reversible inhibition of VRAC. NSAIDs like fenamate improved cognitive function by inhibiting NLRP3 in AD mouse models (Daniels et al. 2016). They are reported as NLRP3 inflammasome inhibitors via suppression of chloride channel (Swanton et al. 2020). Additionally, they can suppress the NF-kB that is responsible for the NLRP3 and pro-IL-1β upregulation (Skokowa et al. 2006).

Bruton tyrosine kinase inhibitors (BTKi): Like NEK, BTK binds to NLRP3 and initiates NLRP3 inflammasome. Studies proved that inhibition of BTK ameliorated NLRP3 activation and improved ischemic brain injury in peripheral blood mononuclear cells (PBMCs) of patients with CAPS (Ito et al. 2015; Mangan et al. 2018). Ibrutinib is a potent small molecule that binds selectively at cysteine 481 residue of BTK and irreversibly inhibits it (Banoth and Cassel 2017). In addition, inhibition of NLRP3 and BTK in sickle cell disease ameliorated the upregulation of platelet aggregation, in mice (Vogel et al. 2021)**.** BTK inhibitors might offer therapeutic benefits in reducing the NLRP3 dependent diseases.

Licochalcone B (LicoB): LicoB is a main bioactive ingredient of licorice that shows anti-inflammatory, antioxidant, and anti-tumor effects (Fu et al. 2013; Wang et al. 2019a). LicoB specifically binds to NEK7 and hampers the interaction among NLRP3 and NEK7, leading to inhibition of NLRP3 inflammasome activation. LicoB eliminates NLRP3 inflammasome activation in macrophages but shows no effect on AIM2 or NLRC4 inflammasome. In mouse models, LicoB exhibited protective role in LPS-induced septic shock, MSU-induced peritonitis, and non-alcoholic steatohepatitis (NASH) via hindering the activation of NLRP3 (Li et al. 2022b).

RRx-001: RRx-001 is a potent and highly specific NLRP3 inhibitor. The bromoacetyl group of RRx-001 covalently binds to cysteine 409 of NLRP3 and subsequently blocks the NLRP3-NEK7 interaction, which is crucial for the formation and activation process of the NLRP3 inflammasome. RRx-001 have shown the capability to suppress the activation of the canonical, noncanonical, and alternative pathways. RRx-001has shown beneficial effects in NLRP3-driven inflammatory diseases such as lipopolysaccharide (LPS)-induced systemic inflammation, DSS-induced colitis and EAE in mice (Chen et al. 2021). Clinical studies showed that RRx-001 exhibited anti-tumour (Morgensztern et al. 2019; Kim et al. 2016b; Carter et al. 2015) and anticancer effects, in phase III clinical studies, with good safety and toxicity profile (Reid et al. 2015). However, its effects on inflammatory conditions remain unknown (Oronsky et al. 2017; Morgensztern et al. 2019).

Tanshinone I (Tan I): Tan I, the main ingredient of Salvia miltiorrhiza, has exhibited anti-inflammatory properties (Liu et al. 2022; Wang et al. 2020a). Tan I works by blocking the NLRP3-ASC connection to prevent the formation and activation of the NLRP3 inflammasome. Tan I had no effect on the activation of the AIM2 or NLRC4 inflammasomes, but suppressed the NLRP3 inflammasome in macrophages. Tan I showed protective effects in mice models of NLRP3 inflammasome-mediated diseases such septic shock and NASH (Zhao et al. 2022).

Luteolin: Luteolin is a flavonoid found in many vegetables and medicinal herbs, which exhibits anti-inflammatory properties in *in-vitro* as well as *in-vivo* models (Xagorari et al. 2001); Chen et al. 2007). Mechanistically, luteolin disrupts the association between NLRP3 and ASC interaction and inhibits NLRP3 inflammasome activation. luteolin supplementation reduced high fat diet induced NLRP3 inflammasome in adipose tissue of ovariectomized mice (M.N. et al. 2021). (Table 1).

**Table 1:** List of NLRP3 inflammasome inhibitors in NLRP3 dependent disease models

NLRP3 inflammasome inhibitors	IC_50_ Value in cells	Disease model	References
MCC950	8nM, BMDMs	APP/PS1 mouse model of AD, Isoflurane-induced cognitive impairment in aged mice PD mice model Experimental TBI mice model Subarachnoid hemorrhage-induced early brain injury in a rat model, intracerebral hemorrhage-induced nerve injury in a rat model EAE is a mouse model of MS HF-induced by pressure overload in obese mice model, HF-induced ventricular arrhythmias Sodium taurocholate-induced acute pancreatitis in rats, Cerulein-induced severe acute pancreatitis in mice model carbon tetrachloride (CCl_4_)-induced acute liver injury	(Dempsey et al. 2017; Fan et al. 2018) (Gordon et al. 2018), (Ismael et al. 2018; Xu et al. 2018). (Luo et al. 2019) (Guo et al. 2022) (Coll et al. 2015; Freeman and Ting 2016) (Wang et al. 2022a; Jiang et al. 2022) (Wang et al. 2022d; Shen et al. 2022). (Yan et al. 2021)
PYD-Only Proteins	-	Macrophages of CAPS patients	(de Almeida et al. 2015)
CY-09	5-6µM, BMDMs	CAPS, Type 2 diabetes mouse model and synovial fluid cells of Gout patients LPS-induced depression in mice High-fat diet-induced NAFLD NAFLD in mice model	(Jiang et al. 2017) (Y. Wang et al. 2022a, b, c, d) (Wang et al. 2021)
OLT1177	1nM, J774A.1	Monocytes of CAPS MSU crystals -induced gouty arthritis APP/PSI mouse model of AD EAE is a mouse model of MS Traumatic spinal cord injury in mice model	(Marchetti et al. 2018a) (Marchetti et al. 2018b) (Lonnemann et al. 2020) (Sánchez-Fernández et al. 2019) (Amo-Aparicio et al. 2022)
Tranilast	25-50µM, BMDMs	CAPS and type 2 diabetes mouse model and synovial fluid mononuclear cells of gout patient Covid-19 patients Genetic gestational diabetes mouse model	(Huang et al. 2018) (Saeedi-Boroujeni et al. 2022) (Saeedi-Boroujeni et al. 2021) (Cao and Peng 2022)
Oridonin	0.5µM,BMDMs	Peritonitis, gouty arthritis, and type 2 diabetes mouse models Trinitrobenzene sulfonic acid-induced colitis mouse model Aβ_1–42_-induced AD in mice Transgenic APP/PS-1 mice model TBI-induced by Closed-head injury using Hall’s weight drop method in mice	(He et al. 2018) (Wang et al. 2015) (Wang et al. 2014) (Zhang et al. 2013) (Yan et al. 2020)
Bot-4-one	0.59-1.28μM, BMDMs and THP-1 cells	BMDMs and Monosodium urate-induced peritonitis mouse model	(Shim et al. 2017)
Parthenolide	5µM, BMDM	LPS-primed primary wild-type BMDM MCAO-induced cerebral ischemia in the rat model Controlled cortical impact (CCI) device-induced TBI in a mouse model, LPS-stimulated BV2 microglia, and HT22 neuron cells stimulated by OGD/R	(Juliana et al. 2010) (Dong et al. 2013) (Ding et al. 2022)
BAY11-7082	5-12µM, BMDMs	LPS-primed primary wild-type BMDMs Imiquimod cream-induced psoriasis STZ-induced diabetic nephropathy in a rat model EAE is a mouse model of MS Nucleus pulposus was implanted in the left L5 dorsal root ganglion (DRG) to mimic Lumbar disc herniation in rats	(Juliana et al. 2010) (Irrera et al. 2017b) (Kolati et al. 2015), (Lang et al. 2022), (Zhang et al. 2017).
MNS	2µM, BMDM	LPS primed BMDMs DSS-induced colitis in a mouse model Pancreatic cancer cell lines SW1990 and PANC-, human pancreatic cancer BALB/c nude mouse model of pancreatic ductal adenocarcinoma	(He et al. 2014) (Zheng et al. 2022) (Liu et al. 2020).
Glyburide	2µM, BMDM	LPS primed BMDMs Hyperoxia-exposed neonatal mice model of bronchopulmonary dysplasia BOO-induced fibrosis in rats Q-VD-OPH induced necroptosis in middle cerebral artery occlusion rat model IL-10-/- mice-induced colitis and Crohn's patients cecal ligation and puncture induced acute liver injury in a mouse model	(Lamkanfi et al. 2009) (Liao et al. 2015) (Hughes et al. 2017) (Hughes et al. 2019) (Teng et al. 2018) (Liu et al. 2017b) (Chen et al. 2016c)
JC-171	10μM, J774A.1	EAE is a mouse model of MS, LPS/ATP in J774A.1 macrophage	(Guo et al. 2017)
BHB	1mM, BMDM	Muckle-Wells syndrome, familial cold autoinflammatory syndrome, and urate crystal-induced peritonitis mouse models, human monocytes Cisplatin-induced acute kidney injury in mice model single prolonged stress-induced PTSD in a rat model chronic unpredictable stress-induced depression in a rat model C6 glioma cells Bovine Peripheral blood mononuclear cells	(Youm et al. 2015) (Luo et al. 2022b) (Yamanashi et al. 2020) (Kajitani et al. 2020) (Shang et al. 2018) (Onizawa et al. 2022).
NSAIDs	50μM, BMDM	LPS primed iBMDMs, 3 × TgAD model and Aβ_1–42_ injection model of AD	(Daniels et al. 2016)
BTKi	-	ischemia/reperfusion-induced brain in mice model PBMCs of X-linked agammaglobulinemia, Muckle-Wells syndrome, and BTK knockout mice model Sickle cell disease mouse model	(Ito et al. 2015) (Liu et al. 2017c) (Vogel et al. 2021)
LicoB	18.1 μM, BMDMs	LPS-induced septic shock MSU-induced peritonitis model methionine- and choline-deficient (MCD) diet-induced NASH model LPS primed BMDMs	(Li et al. 2022b)
RRx-001	116.9 nM, BMDMs	DSS-induced colitis in a mouse model and EAE BMDMs	(Chen et al. 2021)
Tan I	-	LPS-induced septic shock methionine- and choline-deficient (MCD) diet model LPS primed BMDMs	(Zhao et al. 2022)
Luteolin	-	Postmenopausal obesity mouse model Murine macrophage RAW264.7	(M.N. et al. 2021)

*APP*/*PS1* amyloid precursor protein/presenilin 1, *AD* alzheimers disease, *PD* parkinsons disease, *TBI* traumatic brain injury, *EAE* experimental autoimmune encephalomyelitis, *MS* myasthenia gravis, *HF* heart failure, *CAPS* cryopyrin-associated periodic syndrome, *LPS* lipopolysaccharide, *NAFLD* non-alcoholic fatty liver disease, *BMDMs* bone marrow derived-macrophage, *MCAO* middle cerebral artery occlusion, *STZ* streptozotocin, *MNS* 3,4-methylenedioxyβ-nitrostyrene, *DSS* dextran sodium sulfate, *BOO* bladder outlet obstruction, *BBB* Beta-hydroxybutyrate, *PTSD* post-traumatic stress disorder, *BTKi* bruton tyrosine kinase inhibitors, *LicoB* Licochalcone B, *Tan I* Tanshinone I

## Epigenetic mechanisms in the regulation of NLRP3 inflammasome in CNS disorders

The involvement of NLRP3 inflammasome in the pathology of CNS disorders have been established and is being studied widely. It has been shown that the NLRP3 inflammasome is associated with neuritic plaques and significantly higher levels of NLRP3 has been found in the brains of AD patients. By decreasing the Aβ phagocytosis, NLRP3 inflammasome promotes Aβ aggregation in AD (Zhang et al. 2020b; Ising et al. 2019; Li et al. 2023). In human microglia, dopamine inhibits classic inflammasome and α-syn-mediated NLRP3 inflammasome activation. Also, in a mouse model of Parkinsonism, the dopaminergic neurodegeneration and α-syn pathology is related to microglial NLRP3 inflammasome activation (Pike et al. 2022; Fan et al. 2020). It is reported that NLRP3 inflammasome is involved in demyelinating diseases (Martin et al. 2016; Yamamoto et al. 2017). In a mouse model of ALS, microglial NLRP3 inflammation is activated and enhanced the disease progression by increasing caspase 1 and IL-1β levels (Bellezza et al. 2018; Deora et al. 2020). NLRP3 is detected in injured brain that cause inflammatory responses followed by neuronal death in TBI (Ge et al. 2018; Fan et al. 2017). Blocking NLRP3 inflammasome via SIRT3-mediated autophagy improved SCI by reducing mtROS and dyskinesia (Xu et al. 2023; Du et al. 2024). In a rodent model of PTSD, hampering the NLRP3 inflammasome reduced anxiety behaviour (Yamanashi et al. 2020; Govindula et al. 2023). In major depressive disorder patients, NLRP3 is activated and enhanced IL-1β and IL-18 levels in serum (Alcocer-Gómez et al. 2014). Thus, blockade of NLRP3 inflammasome could represent a potential target in the treatment of CNS disorders.

Epigenetic changes are reversible changes in gene expression without altering the DNA sequences. The identified epigenetic changes are DNA methylation, Histone modification, and Non-coding RNAs alterations (Poli et al. 2020). Epigenetic mechanisms are correlated with NLRP3 inflammasome in several neuropathogenic conditions. Consequently, targeting the epigenetic mechanisms shows therapeutic benefits in NLRP3-mediated CNS disorders.

DNA methylation: DNA methylation is a crucial epigenetic mechanism that involves the addition of a methyl group to the fifth position of cytosine residues in CpG dinucleotides. This change, known as DNA methylation, results in transcriptional repression and is important in many processes, including development, ageing, and cancer (Greenberg and Bourc’his 2019). DNA methylation is necessary for normal cell activity, but it is also linked to the origin of several disorders. It is confirmed that patients with CAPS and familial Mediterranean fever (FMF) syndromes show higher expression of IL-1β and are related to the demethylation of NLRP3 inflammasome in monocytes. In patients treated with IL-1β antagonists methylation levels were restored, suggesting that regulating the methylation of NLRP3 inflammasome components is crucial. Also, the demethylation of ASC is correlated with enhanced tumor size in glioblastoma (Martinez et al. 2007). A study showed that NLRP3 DNA methylation levels are strongly correlated with cortical thickness in several areas of the brain in MDD patients in comparison to healthy controls (Han et al. 2022).

Histone modifications: Histones are essential proteins that wrap DNA into nucleosomes, the chromatin-building blocks. At their N-terminal tails, these histones undergo a variety of post-translational modifications (PTMs), including phosphorylation, acetylation, methylation, and others (Farrelly et al. 2019). These changes are controlled by chromatin remodelling enzymes, which influence chromatin shape and govern its accessibility for transcriptional expression (Tessarz and Kouzarides 2014). Dysregulation of these enzymes can lead to pathological conditions. In essence, the interaction of histone PTMs with chromatin remodelling enzymes is critical in regulating gene expression and cellular function (Bhaumik et al. 2007). Several inflammatory models are used to study the impact of histone epigenetic alteration (Bayarsaihan 2011).

In a murine model, administration of bortezomib, a proteasome inhibitor, in dorsal root ganglion induced painful neuropathy by phosphorylating signal transducer and activator of transcription-3 (STAT3) and acetylation of histone H3 and H4 in the NLRP3 promoter region. Bortezomib-induced painful neuropathy is minimized when NLRP3 expression is blocked (Liu et al. 2018). Aged C57BL/6J mice exposed to sevoflurane-induced cognitive impairment and upregulated NLRP3 inflammasome by inactivation of autophagy processes. However, treatment with Suberoylanilide hydroxamic acid (SAHA) a histone deacetylase (HDAC) inhibitor, activated autophagy and downregulated NLRP3 inflammasome by enhancing the H3 and H4 acetylation. This shows that histone acetylation activates autophagy which plays a central role in reducing neuroinflammation by inhibiting NLRP3inflammasome (Fang et al. 2021). Administration of β-Hydroxybutyrate (BHB), a specific class I HDAC inhibitor, in the murine model of AD restricted plaque formation by decreasing the NLRP3 inflammasome and BHB level is reported to be lower in the brain parenchyma and red blood cells of AD patients, suggesting the importance of BHB in AD pathology (Shippy et al. 2020). Furthermore, mice treated with HDAC inhibitor, sodium butyrate regained their learning ability as well as long-term memory (Fischer et al. 2007). In the AD of humans and a mouse model, elevated HDAC 2 expression is observed (Gonzalez-Zuñiga et al. 2014; Liu et al. 2017a). In a murine model of PD, the presence of histone 3 lysine 27 trimethylation (H3K27me3) repressive mark in nuclear factor-erythroid 2-related factor 2 (Nrf2) promoter region suppressed its expression and enhanced inflammasome associated proteins such as NLRP3, ASC, cleaved caspase 1 as well as ROS generation (Cai et al. 2020). Notably, HDAC 2 enzyme deacetylates histone substrates at the promoter region of several synaptic-plasticity-associated genes, reducing memory function and synaptic plasticity (Guan et al. 2009). HDAC 2 plays a crucial role in the maturation of synapses (Akhtar et al. 2009). Mithramycin A significantly arrested HDAC 2 gene and protein expression, resulting in the recovery of synaptic plasticity gene expressions in SH-SY5Y cells overexpressed with amyloid precursor protein (Subba et al. 2020). A study reported that decreasing STAT3 phosphorylation reduced H3 and H4 acetylation on the promoter region of NLRP3 resulting in lower NLRP3 inflammasome expression in ischemic stroke injury (Zhu et al. 2021).

Noncoding RNAs (NcRNAs): ncRNAs are a type of RNA transcript that do not encode proteins. They can exhibit epigenetic modulation by remodelling chromatin or affect gene expression at the transcriptional or post-transcriptional levels (Kaikkonen et al. 2011). Their abnormal expression contributes to the onset and progression of CNS diseases. The root cause of their abnormal expression can be related to epigenetic changes (Kumar et al. 2020). Evidence suggests that NLRP3 inflammasome activation could be controlled by ncRNAs (Feng et al. 2021). ncRNAs are expressed differently in a variety of CNS diseases associated with neuroinflammation. Recognising how these ncRNAs regulate NLRP3 inflammasome will provide valuable therapeutic strategies in preventing CNS diseases (Yang et al. 2023).

NcRNAs are divided into miRNAs and long non conding RNAs (lncRNAs). MiRNAs are conservative, single-stranded endogenous non-coding RNAs that range in length from 19 to 24 nucleotides. generally, miRNAs are produced from hairpin-shaped transcripts and incorporated into the argonaute protein as part of a silencing complex (Tezcan et al. 2019; Bishop 2004). miRNAs' has the ability to regulate the expression of histone modifications, including histone deacetylases and DNA methyltransferases (Fabbri et al. 2007).

**Fig. 3:**
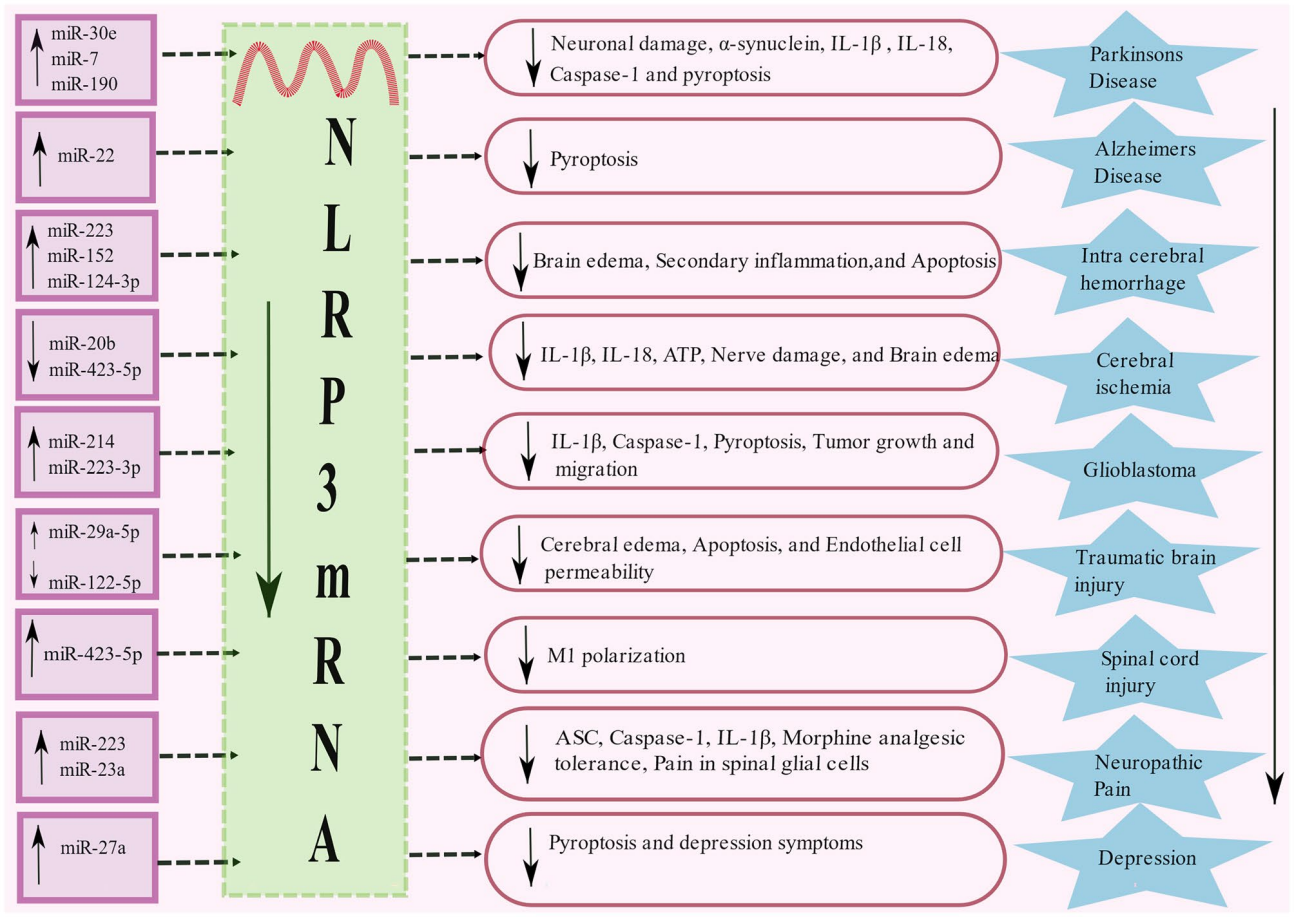
List of mRNAs modulating NLRP3 in CNS disorders: miRNA interacts with the UTR region of NLRP3 mRNA and inhibits protein translation. Inhibition of the formation of NLRP3 protein ameliorates the assembly of NLRP3 inflammasome. Upregulation of multiple miR-30e, miR-7, miR-190, miR-22, miR-223, miR-152, miR-124-3p, miR-214, miR-223-3p, miR-29a-5p, miR-423-5p, miR-23a, miR-27a and downregulation of miR-20b, miR-423-5p, miR-122-5p suppresses the NLRP3 mRNA and prevent the development of PD, AD, Intracerebral hemorrhage, Cerebral ischemia, Glioblastoma, TBI, SCI, Neuropathic pain, and Depression

MicroRNA expression: miRNAs regulate post-transcriptional repression or mRNA degradation (Poli et al. 2020). The release of miRNAs is crucial for the growth and function of the CNS. Subsequently, its disruption is implicated in CNS disease pathologies (Wang et al. 2012). NLRP3 activity is regulated by several miRNAs, and it has a conserved binding site for miRNA in its 3’-untranslated region (UTR) region. The interaction between this conserved region of 3’UTR of NLRP3 and miR-30e reduces NLRP3 activity by interfering with its protein translation and improves neuronal damage in mice MPTP model of PD (Li et al. 2018). Similarly, miR-7 which is abundant in neurons inhibits α-synuclein protein levels via 3’-UTR of α-synuclein mRNA (Junn et al. 2009). Also, miR-7 and miRNA-190 regulate neuroinflammation in Parkinson's disease by binding to NLRP3 inflammasome in a mouse model (Zhou et al. 2016b; Sun et al. 2019). Moreover, exogenous administration of miR-30e, miR-190, miR-7 suppresses the generation of IL-1β and IL-18 in PD by inhibiting NLRP3, ASC, caspase-1 protein levels (Li et al. 2018; Zhou et al. 2016b; Sun et al. 2019) (Fig 3). Notably, miRNA-135b protects against PD by modulating FoxO1-induced NLRP3 inflammasome and pyroptosis in *in-vitro* (Zeng et al. 2019)*.*Increased expression of miR-223-3p and mir-7-5p does not decrease the expression of NLRP3 inflammasome and IL-1 and IL-18 production significantly in LPS primed and Aβ42-stimulated PBMC of AD patients (La Rosa et al. 2021). miRNA-22 mimics improved memory ability in AD via suppressing pyroptosis and NLRP3 inflammasome (Han et al. 2020). Furthermore, in intracerebral hemorrhage (ICH) animal models, miR-223 and miR-152 repressed NLRP3 inflammasome activation that resulted in a decrease in brain edema and enhanced neurological function (Yang et al. 2015; Hu et al. 2020). Besides, in the basal ganglia of ICH patients, miR-124-3p arrested the secondary inflammation in microglia by hampering NLRP3 inflammasome activation via TRAF6 (Fang and Hong 2021). Conversely, the downregulation of miRNA-20b reduced IL-1β and IL-18 levels, ATP, and ROS by repressing the NLRP3 pathway in cerebral ischemia (Zhao et al. 2019).

In glioblastoma, the post-transcriptional regulators of NLRP3 such as miR-22 and miR-30e are reported to be under-expressed (Chen et al. 2016a; Chakrabarti et al. 2016). miR-223 was found to be less in glioblastoma and overexpression of miR-223 arrested NLRP3 inflammasome contributing to the repression of cell proliferation and migration (Ding et al. 2018). It was also observed that miR-214 inhibits the caspase-1 and NLRP3 expression resulting in the suppression of tumor growth and migration in glioblastoma (Yang et al. 2022). miR-29a-5p mimics reported a protective role in TBI via targeting the NLRP3 pathway and alleviated BBB dysfunction and cerebral edema in a mouse model (Zhang et al. 2021a). In addition, inhibition of miR-122-5P alleviates microglial inflammation and apoptosis in TBI by promoting microglial polarization shift M1 to M2 phenotype and suppressing the NLRP3 inflammasome signaling (Kang et al. 2022). Enhanced expression of miR-423-5p inhibited LPS-induced M1 polarization by targeting NLRP3 expression in SCI (Cheng et al. 2021). Despite upregulation, the knockdown of miR-423-5p in rats improved brain water content and nerve damage in cerebral ischemia and inhibited NLRP3 inflammasome activation (Luo et al. 2022a).

In a neuropathic pain rat model, miR-223 relieved morphine analgesic tolerance by suppressing NLRP3 inflammasome (Xie et al. 2017). Overexpression of miR-23a in spinal glial cells controlled neuropathic pain by targeting TXNIP/NLRP3 inflammasome axis (Pan et al. 2018). In the mice model of depression, miR-27a ameliorated NLRP3-mediated pyroptosis via SYK/NF-κB axis (Li et al. 2021b). In mice, intravenous administration of miR-223-3p hampered the NLRP3 inflammasome pathway in Streptococcus equi subsp. zooepidemicus (SEZ) infection. SEZ is an important pathogen that causes a wide variety of infections, particularly meningitis, endocarditis, and septicemia. Therefore, overexpression of miR-223-3p is needed to protect against SEZ infection (Li et al. 2022a). These findings show the significance of miRNA-based treatments for CNS illnesses associated with NLRP3 inflammasome.

Long non-coding RNAs: lncRNAs, which are around 200 nucleotides lengthy play a variety of roles in biological functions, including regulating DNA synthesis, transcription, and protein translation (Jiang et al. 2019). Additionally, they have been linked to a variety of physiological and clinical events (Flynn and Chang 2014; Batista and Chang 2013). LncRNAs interact with proteins, miRNAs, and DNA through interaction domains, using the benefits of their secondary structures and sequences to perform regulatory functions. Mounting evidence emphasizes the critical function of lncRNAs in controlling the NLRP3 inflammasome's activity in various disease states. On the nuclear and cytoplasmic levels, lncRNAs intricately regulate the NLRP3 inflammasome's activity, affecting chromatin structure, gene transcription, and translation (Menon and Hua 2020; Zhang et al. 2019; Mathy and Chen 2017; Fernandes et al. 2019). The lncRNA EPS suppresses the generation of the ASC adaptor protein, blocking the activation of the NLRP3 inflammasome in resting macrophages (Lu et al. 2016). lncRNA Gm15144 suppress the activation of NLRP3 inflammasome and further generation of caspase-1 and IL-1β, by blocking the TXNIP during fasting. Also, knockdown of Gm15144 in mouse model enhanced the TXNIP, caspase-1 and IL-1β in hepatic inflammation (Brocker et al. 2020). In contrast, LncRNA MALAT-1 and LncRNA neat-1 enhanced the activation of NLRP3 inflammasome (Han et al. 2018; Zhang et al. 2019). Therefore, understanding the mechanisms behind lncRNA modulation of NLRP3 inflammasome sustains the targets in treatment of inflammatory diseases.

## Prospects and Conclusion

Several lines of scientific evidence suggest that neuroinflammation is a principal pathological feature of CNS disorders. An essential component of neuroinflammatory involvement; is NF-kB followed by NLRP3 activation (Welcome 2020; Chen et al. 2016b). As a result, targeting and prevention of neuroinflammation by inhibition of NF-kB and NLRP3 could potentially provide a novel therapy for CNS disorders. In this regard, it is necessary to focus on the priming signal, the initial step in the NLRP3 activation by the NF-kB pathway. Several studies showed that NF-kB is involved in inflammatory pathways. So, developing NF-kB inhibitors may prevent inflammatory diseases. Although, researchers developed NF-kB inhibitors their clinical evidence is limited. NLRP3 stimuli such as PAMPs, DAMPs, ionic flux, lysosomal disruption, Mitochondrial dysfunction, and ROS enhance the NLRP3 inflammasome signaling but how these stimuli are regulated in particular cases are need to be understood in a detailed manner (Pellegrini et al. 2019). Evidence suggests that targeting the NLRP3 inflammasome upstream as well as downstream stimuli can impact NLRP3 inflammasome activity and expression (Alishahi et al. 2019). These findings help to create a new therapeutic option for the hampering NLRP3 inflammasome. Currently, NeK7 has been identified as an NLRP3 inflammasome promotor. However, underlying mechanisms remain to be understood and investigated.

The activated NLRP3 inflammasome is crucial for stimulating caspase-1 which further leads to secondary inflammation followed by neuronal damage (Song et al. 2017) Nonetheless, targeting the caspase-1 might indirectly help to prevent the neuronal damage. Also, pyroptosis is being emerged as a crucial step in inflammatory diseases. Hence, inhibition of GSDMD serves as an anti-inflammatory effect (Kanneganti et al. 2018). Moreover, considering the mechanisms of early stages of NLRP3 inflammasome activation such as sensor, adaptor, and effector binding helps in the regression of NLRP3-mediated inflammatory diseases. Importantly, targeting the structure and assembling of the NLRP3 inflammasome could ultimately help to suppress the NLRP3 inflammasome stimulation and supports a better treatment for inflammatory and neurodegenerative disorders. Recent research has resulted in the development of specific and non-specific NLRP3 inflammasome inhibitors that have proven their efficacy in *in-vivo* and *in-vitro* studies of NLRP3-mediated diseases. However, their clinical efficacy is still under evaluation. Currently, Food and Drug Administration (FDA) approved IL-1 receptor inhibitors canakinumab, rilonacept, and anakinra as safe and effective in treating NLRP3-driven diseases. Future studies are needed in developing, analyzing, and verifying the NLRP3 inflammasome antagonists for clinical use. Moreover, research on NLRP3 inhibitors in CNS disease is currently lacking. As a response, strategies must be developed to disentangle neural dysregulation.

The epigenetic studies in NLRP3 modulation give excellent evidence and optimism for future research and therapy options for CNS disorders via modification of the NLRP3 inflammasome pathway (Poli et al. 2020). Reducing the overexpression of NLRP3 inflammasome elements and decreasing their stabilization restricts the inflammasome assembly and activation (Raneros et al. 2021). Moreover, research on the regulation of DNA methylation and histone modifications of NLRP3 inflammasome components is lacking. MicroRNAs are critical regulators that have been connected to the expression and control of various genes in CNS disorders and are widely expressed in neurons which leads to interest in CNS disorders. Although NLRP3 inhibitors and epigenetic pathways have been successfully studied in animal model research, they are yet to be employed in clinical settings. Further, clinical studies need to be conducted to attest to the function of NLRP3 inhibitors and epigenetic modulators in NLRP3-driven diseases.

## Data Availability

Not applicable.
